# Pharmacology of Hallucinations: Several Mechanisms for One Single Symptom?

**DOI:** 10.1155/2014/307106

**Published:** 2014-06-04

**Authors:** Benjamin Rolland, Renaud Jardri, Ali Amad, Pierre Thomas, Olivier Cottencin, Régis Bordet

**Affiliations:** ^1^Department of Addictionology, CHU Lille, 59037 Lille, France; ^2^Department of Pharmacology, Univ Lille Nord de France, EA 1046, 59000 Lille, France; ^3^Functional Neurosciences & Disorders Laboratory, Université Lille Nord de France, EA 4559, 59000 Lille, France; ^4^Department of Pediatric Psychiatry, CHU Lille, 59037 Lille, France; ^5^Department of Psychiatry, CHU Lille, 59037 Lille, France

## Abstract

Hallucinations are complex misperceptions, that principally occur in schizophrenia or after intoxication induced by three main classes of drugs: psychostimulants, psychedelics, and dissociative anesthetics. There are at least three different pharmacological ways to induce hallucinations: (1) activation of dopamine D2 receptors (D2Rs) with psychostimulants, (2) activation of serotonin 5HT2A receptors (HT2ARs) with psychedelics, and (3) blockage of glutamate NMDA receptors (NMDARs) with dissociative anesthetics. In schizophrenia, the relative importance of NMDAR and D2R in the occurrence of hallucinations is still debated. Slight clinical differences are observed for each etiology. Thus, we investigated whether the concept of hallucination is homogenous, both clinically and neurobiologically. A narrative review of the literature is proposed to synthesize how the main contributors in the field have approached and tried to solve these outstanding questions. While some authors prefer one explanatory mechanism, others have proposed more integrated theories based on the different pharmacological psychosis models. In this review, such theories are discussed and faced with the clinical data. In addition, the nosological aspects of hallucinations and psychosis are addressed. We suggest that if there may be common neurobiological pathways between the different pharmacological systems that are responsible for the hallucinations, there may also be unique properties of each system, which explains the clinical differences observed.

## 1. Introduction

A hallucination is a type of misperception that can be defined as “the perception of an object without an object to perceive” [1]. While hallucinations may occasionally occur in diverse psychiatric and neurological pathologies, they are particularly characteristic of schizophrenia-related disorders, in which antipsychotic drugs are commonly used to treat them. However, hallucinations may also be triggered by at least three different kinds of drugs: psychostimulants (i.e., cocaine or amphetamine), the so-called “dissociative anesthetics” (i.e., phencyclidine (PCP) or ketamine), and psychedelics, (i.e., lysergic diethylamid (LSD) and psilocybin).

Depending on which situation is considered, the pharmacological hypotheses underlying the symptoms are completely different. Psychostimulants-induced hallucinations result from increased dopamine transmission and hyperactivation of dopamine D2 receptor (D2R). Furthermore, “dissociative anesthetics” drugs induce complex schizophrenia-like clinical pictures, including hallucinations, that result from the blockade of glutamate NMDA receptors (NMDAR). Lastly, psychedelics act by stimulating the serotoninergic 5HT2A receptor (5HT2AR). In schizophrenia, although antipsychotic blocking studies suggest that hallucinations result from D2R hyperstimulation, there are also numerous arguments for NMDAR dysfunction, which may be a potential and specific hallucinatory mechanism.

Initially, the existence of these different pharmacological systems underlying hallucinations appears incompatible with a unified conception hallucination. It is necessary to articulate these three mechanisms into an integrated model, or, alternatively, there may be different forms of hallucinations, that are mediated by different pharmacological supports and neurobiological circuits.

## 2. The Three Main Pharmacological Models of Hallucinations

Three different types of psychoactive drugs can induce hallucinations in humans. In each case, a specific pharmacological process is involved (Table 1). Psychostimulants stimulate D2Rs, dissociative anesthetics block NMDARs, and psychedelics stimulate H5T2ARs. In schizophrenia, both D2Rs and NMDARs are involved. In each type of situation, slight clinical features are observed (Table 1).

### 2.1. The Dopamine Model: Hallucinations, Antipsychotics, and Schizophrenia 

Schizophrenic hallucinations are mainly auditory verbal [2, 3]. However, notable exceptions include early-onset forms in which visual and multisensory hallucinations are more frequent [4]. Schizophrenic hallucinations combined with other psychotic symptoms are commonly classified within the “positive symptoms” of schizophrenia [5]. It is these positive symptoms on which antipsychotic drugs have the most blatant therapeutic effects [6], and successive studies from the 1960's revealed that this effect could be due to antagonistic action on D2Rs, which is shared by all antipsychotic molecules [7]. Consequently, the hypothesis that positive symptoms may be related to an excessive transmission of dopamine has become the main pharmacological model of positive symptoms in schizophrenia [5]. This hypothesis has been reinforced by contemporaneous imaging techniques, which have confirmed that positive symptoms were associated with an increase of dopaminergic activity in the striatum [8]. For a while, the other dopamine receptors, notably the D1 receptors, were suggested as other receptors possibly implicated in positive symptoms of schizophrenia [9], but it appeared that they were probably more involved in negative symptoms of schizophrenia, which consist of blunted effects and social withdrawal [10, 11]. D2Rs thus emerged as the primary dopaminergic modulators underlying positive symptoms [7].

It has secondarily been hypothesized that all forms of D2R overstimulation in striatum could trigger psychosis, even beyond the scope of schizophrenia. The clinical picture of psychosis induced by psychostimulant drugs [12], which result from sustained dopamine action [13], appears relevant to this hypothesis [14]. Psychotic symptoms can also result from the use of dopaminergic receptors agonists in Parkinson's disease [15]. Thence, it has been suggested that striatal dopaminergic hyperfunction may better fit with psychosis than with schizophrenia, as nonpsychotic forms of schizophrenia are not linked with such striatum-based anomalies [7]. Psychotic symptoms in schizophrenia may be related to a neurobiological pathological process, incentive salience, which is the cognitive consequence of dopaminergic-enhanced transmission, and may pave the way for the emergence of psychosis [7].

It has thus been proposed that dopamine is the pharmacological keystone of all psychotic states, which is the expression of a process of dopamine supersensitivity, because of an increase in the high-affinity states of the D2Rs, or D2High receptors [16]. According to this theory, the clinical activity of the hallucinogenic drugs may result from the property of these drugs to target D2High receptors, and this action may be the fundamental pharmacological mechanism underlying psychosis [17]. This “all-dopamine” conception of psychosis will be discussed below.

### 2.2. The Glutamate Model: Hallucinations, Dissociative Anesthetics, and Schizophrenia

Shortly after the synthesis of a new class of anesthetic drugs at the end of the 1950s, it was observed that these drugs could induce schizophrenia-like symptoms, with a combination of hallucinations, negative symptoms, and dissociative symptoms. These drugs were consequently known as “dissociative anesthetics” [18]. The main molecules of this class are phencyclidine (PCP) and ketamine.

It has been demonstrated that PCP exhibits antagonistic effects on NMDARs [19]. As a result, a new pharmacological model of hallucinations and other schizophrenic symptoms was introduced [20, 21]. Subsequently, a progressive amount of evidence indicated that many susceptibility genes for schizophrenia were related to the functioning of NMDARs [20, 21] and that glutamate may have a more central place in the pathophysiology of schizophrenia than dopamine [22]. The role of NMDAR in schizophrenia was also highlighted by the effectiveness of several NMDAR regulators on both positive and negative symptoms of schizophrenia [23]. All these arguments have progressively led to an increased interest in the role of NMDARs concerning the whole pathophysiology of schizophrenia [24]. Currently, NMDAR hypofunction is considered by several leading researchers of the field to be a major neurobiological hypothesis for schizophrenia [25].

Apart from the use of PCP or ketamine, other nonschizophrenic NMDAR-related psychoses have been reported [26]. NDMAR-related psychosis is thus not confined to the spectrum of schizophrenia. Moreover, the sole blockade of NMDAR, without any relation with the dopaminergic system may be sufficient to induce psychosis [21]. In these types of states, mixed positive and negative symptoms are observable, which is in contrast to what happens with the use of dopaminergic drugs. As these states also include hallucinations, it could be concluded that purely NMDAR-related hallucinations are conceivable, without any relation with the dopaminergic system.

### 2.3. The Serotonin Model: Hallucinations, Psychedelics, and Schizophrenia

Psychedelics constitute a heterogeneous class of molecules, among which LSD and psilocybin are the two most well-known and best studied molecules [27]. Psychedelics induce phenomenologically complex pictures, which can mix visual hallucinations, synesthesia, and peculiar altered states of consciousness with mystical feelings [28]. Although there has been much debate regarding the psychedelics' exact pharmacological mechanism [29], the most commonly admitted mode of activity of this class of drugs is the stimulation of serotonin 5HT2AR on cortical neurons [30, 31]. Cortical 5HT2AR hyperactivation may affect the functioning of the cortico-striato-thalamo-cortical loops and triggers a disruption in the thalamic gating of sensory and cognitive information [28]. It has been proposed that this process triggers a breakdown of cognitive integrity and results in the subsequent occurrence of aberrant feelings and perceptions [28].

As soon as the serotoninergic-based mode of action of LSD was discovered, a serotoninergic hypothesis of schizophrenia was proposed, prior to being supplanted by the dopaminergic hypothesis [32]. Today, several authors have proposed a reappraisal of the role of the 5HT2AR in both schizophrenia and psychosis [28, 31]. However, psychedelics-induced forms of psychosis sensibly differ from schizophrenia-like psychosis, in particular regarding the clinical aspect of hallucinations. Visual hallucinations are typical with psychedelics, whereas auditory hallucinations are much more rare [33]. Furthermore, “pseudohallucinations” (i.e., misperceptions with intact reality testing and insightfulness) are very frequent [33], although insight into hallucinations in schizophrenia is quite poor [34].

Despite these numerous clinical differences, there are some arguments that suggest a role of 5HT2AR in schizophrenia. The level of expression of 5TH2AR is upregulated in young and untreated patients with schizophrenia, and because visual hallucinations frequently occur in the early phases of schizophrenia, it has been proposed that psychedelic-induced pictures may be related to early forms of schizophrenia [31]. Furthermore, many second-generation antipsychotics have an antagonistic action on the 5HT2AR, which highlights the role of 5HT2ARs in schizophrenia [35]. However, the antipsychotic action of this antagonist effect remains questionable and will be discussed in the next chapter.

Consequently, it appears that some specific forms of hallucinations may result from 5HT2AR activation, in addition to other specific clinical abnormalities. At first glance, these particular clinical pictures do not appear to be linked with the participation of NMDARs or D2Rs.

## 3. Confrontations between Models and Attempts for Integration

Because three different cerebral receptors contribute to the triggering of different hallucinatory processes, it is necessary to assess whether these three receptors are part of the same global neural circuitry, which would preserve the conceptual unity of hallucinations from a pharmacological perspective, of whether they belong to pathways that induce hallucinations separately, which would mean that there are several pharmacological forms of hallucinations, with each having specific clinical expressions. This has led scientists to question each model in front of the two other ones. We will first focus on discussions of two-system interactions (NMDAR-D2R, 5HT2AR-D2R, and 5HT2AR-NMDAR) and then move to theories that attempt to integrate all of the three receptors.

### 3.1. Glutamate/Dopamine Interactions

Glutamate and dopamine hypotheses of schizophrenia may be considered as rival models, particularly in regards to the pathophysiology of positive symptoms in schizophrenia. Several experts believe that one of the models is more superior than the others. According to the supporters of the “all-dopamine” hypothesis, the role of dopamine is central, and the schizophrenia-like effects of ketamine and PCP may be at least partially explained by the affinity of the drugs to the D2High receptors [7]. In this hypothesis, activation of D2Rs is indispensable to induce any form of psychosis [16] and consequently any form of hallucinations.

However, other authors note that psychostimulants (i.e., pure dopaminergic drugs) are far less hallucinogenic compared with dissociative anaesthetics [36] and that many symptoms induced by NMDAR antagonists have been reported not to be linked with an increase of dopamine transmission in the striatum [21]. Moreover, in animal models, only few antipsychotic drugs can reverse the effects of acute and chronic administration of PCP on prepulse inhibition [37], which is a cognitive parameter used as a model of positive symptoms [38]. NMDAR blockade is thus considered as an independent mechanism for the induction of psychosis [24].

Interconnections and reciprocal regulations between the two systems are also possible. The prefrontal cortex may trigger NMDAR-mediated decrease of the dopaminergic tone in striatum [39]. Moreover, dopaminergic and glutamatergic systems may have opposite effects in the striatum, which may explain that both D2R activation and NMDAR blockade induces hallucinations in a similar manner [36]. Animal studies appear to validate this hypothesis, as NMDAR modulation limits some behavioural effects induced by amphetamine [40]. However, this may also indicate a two-way interaction, as recent studies have reported that activation of D2Rs induces a multimodal downregulation of NMDAR in the striatum [40], resulting in reciprocal regulations between the dopaminergic and glutamatergic systems. As discussed below, the role of 5HT2ARs has been suspected for its involvement within this complex neural circuitry.

D2R-NMDAR interactions in the striatum still remain to be confirmed. However, recent studies question the potential influences of D2Rs on ketamine-induced abnormalities in the striatum [41]. Whether the dopaminergic and the glutamatergic systems function jointly or separately in striatum remains a key issue toward understanding the ability of both NMDARs and D2Rs modulators in inducing or reducing hallucinations.

### 3.2. Dopamine/Serotonin Interactions

The role of 5HT2ARs has been recently reintroduced in schizophrenia, since many second-generation antipsychotics are both D2R and 5HT2AR antagonists [35]. This suggests that the blockade of 5HT2AR may underlie the antipsychotic effects of these drugs, in accordance with the 5HT2AR-mediated hallucinogenic properties of psychedelics [31]. However, second-generation antipsychotics are characterized by the triggering of lesser side effects that result from the blockade of D2Rs in the non-limbic areas, such as extrapyramidal symptoms or galactorrhea [35]. This observation has led to the hypothesis that 5HT2AR blockade reverses the effects of D2R blockade only in these areas [42], whereas the D2R antagonistic effects of second-generation antipsychotics are preserved in the limbic system, which preserves the therapeutic activity of these drugs on positive symptoms of schizophrenia [35]. Consequently, it is not obvious that the blockade of 5HT2ARs is responsible for the effects of these drugs on positive symptoms. Furthermore, first generation antipsychotics, which have a lack or little activity on 5HT2ARs, exhibit the same level of efficacy on the positive symptoms compared with more recent drugs [6, 43].

However, several studies have investigated the D2R-related theory of positive symptoms in schizophrenia and the activity of psychedelics. As previously described, some researchers have justified this issue with the idea that psychedelics function because of their stimulating effect on D2Rs [17]. Several studies have supported this notion. For example, LSD exhibits biphasic activity in mice; the first phase involves only 5HT2ARs, and the second phase, which is related to the psychotic symptoms observed in humans, involves only D2R [44, 45]. Nevertheless, according to other studies, psychedelic-induced stimulation of 5HT2ARs in the prefrontal cortex is responsible for a downstream activation of dopaminergic neurons that are located in the ventral tegmental area [46]. Moreover, the formation of heteromers involving both 5HT2AR and D2R has been observed on the membranes of mouse striatal neurons, which may result in a functional crosstalk between the two neurotransmission systems [47]. In this theory, however, the 5HT2ARs implicated in psychosis are located in the striatum and not in the prefrontal cortex, which suggests that they interact with D2Rs.

Yet, other arguments support that psychedelic-induced hallucinations are not related to the modulation of D2Rs. Haloperidol was found unable to block the psychotomimetic effects of psilocybin, whereas the 5HT2AR antagonist ketanserin was able to do so, notably regarding VH [48, 49]. Consequently, it remains very unclear whether 5HT2ARs and D2Rs may interact in schizophrenia hallucinations if D2Rs are not involved in psychedelic-induced hallucinations.

### 3.3. Glutamate/Serotonin Interactions

Both NMDAR antagonists and 5HT2AR agonists induce hallucinations and have been used in drug-induced experimental models of schizophrenia. In addition, some specific cognitive functions, such as the inhibition of return, are impaired in schizophrenia and are disrupted with both NDMAR antagonists and HT2AR agonists [50]. However, other cognitive impairments involved in schizophrenia, including deficits in mismatch negativity, are only reported when administering NDMAR antagonists [51]. Moreover, the prepulse inhibition of the acoustic startle reflex, which is reduced in schizophrenic patients, appears to be increased only by NMDAR antagonists and is unmodified following administration of 5HT2AR agonists in humans [52]. Clinically, NDMAR antagonists also induce negative symptoms, and this class of drugs may present a more sustained face of validity with schizophrenia than psychedelics.

Nevertheless, several attempts for integrating NMDAR and 5HT2AR into a common neurobiological framework for psychosis have been proposed. According to some of these theories, abnormalities in one of the two neurotransmission systems could trigger dysfunctions in the other. For example, noncompetitive antagonists NMDAR appear to potentiate the activation of serotoninergic receptors [53], while positive modulators of NMDARs could inhibit serotoninergic activation [54]. Thus, psychosis may be at least partially the expression of mechanisms in series, in which NMDAR dysfunction leads to the enhanced activation of 5HT2ARs [53]. Other models hypothesize that psychosis results from a final common pathway that is equally disrupted by both NMDAR hypoactivation or 5HT2AR hyperactivation. For example, it is thought that an impairment in the cognitive function of inhibition of return is responsible for the occurrence of psychosis and results from dysfunctions in several different psychopharmacological pathways, that is, NDMAR blockade or 5HT2AR stimulation [50].

More recently, different types of interconnections between glutamatergic and serotoninergic neurotransmission systems have been proposed to explain psychosis. Recent work has shown that the action of psychedelic drugs on 5HT2ARs requires the indispensable formation of a complex between 5HT2AR and the metabotropic glutamatergic mGlu2 receptor (mGlu2R) [55]. Furthermore, mGlu2R and mGlu3R agonists experimentally reverse the effects of NMDAR antagonist drugs [56] and appear to have antipsychotic effects in human, notably on positive symptoms of schizophrenia [57]. This suggests that a common pharmacological process involving mGlu2R and mGlu3R hypoactivation may be the missing link between the clinical activities of both NMDAR antagonists and 5HT2AR agonists [58]. The role of dopamine and the mode of action of current antipsychotic drugs are still unclear in relation to this theory, and attempts at a more unified theory would require a multipharmacological model.

### 3.4. NMDAR/DR2/5HT2AR Interactions

Because the three pharmacological systems described above that obviously have close interactions between each other, it could be assumed that they belong actually to a complex and integrated neurobiological circuit, whose impairment could occur at various levels in case of psychosis. Experimental studies have shown that the three different systems appear interdependent in inducing psychosis-like behavioral abnormalities in rodents [53, 59]. Recently, several leaders of the field have proposed an entirely integrated model that includes several different neurotransmission systems, most notably the three that are discussed here [28].

**Figure 1:**
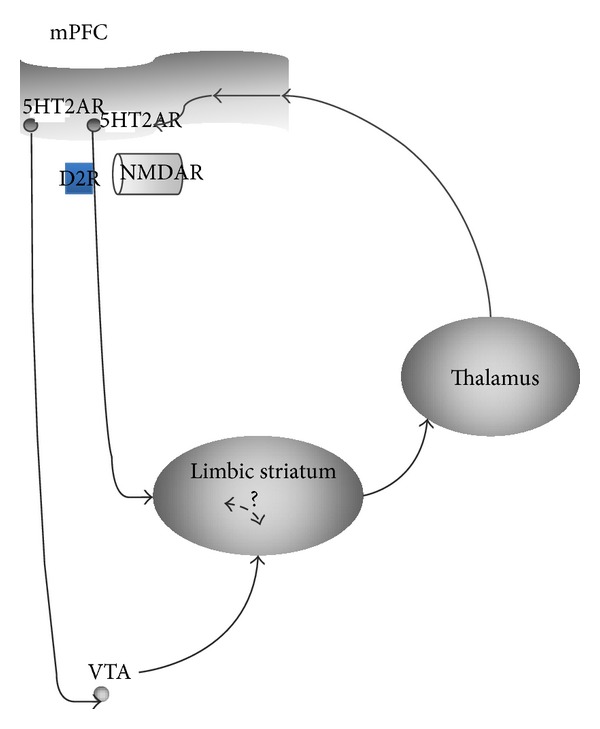
5HT2AR/D2R/NMDAR interactions. Simplified version of the Geyer and Vollenweider model of psychosis [25], which supposes a disruption in the cortico-striato-thalamo-cortical loops. This model tries to connect 5HT2R, D2R, and NMDAR in a unified neurobiological system which could be impaired in psychosis.* Abbreviations*. mPFC: medial prefrontal cortex; VTA: ventral tegmental area; NMDAR: N-methyl-D-aspartate receptor; D2R: dopamine-2 receptor; 5HT2R: 5-hydroxytryptamine-2A receptor.

This model is constructed around the hypothesis that psychotic symptoms could result from filtering disruptions within the cortico-striato-thalamo-cortical loops, which underlie the level of awareness and attention that is dedicated at any time in the brain to an external stimulus [28] (Figure 1). By disorganizing the filtering processes, different kinds of drugs could trigger psychotic symptoms, and any of D2Rs, 5HT2ARs, and NMDARs may constitute unspecific vulnerability points in this circuitry. D2Rs and NMDARs act directly in the limbic striatum, whereas prefrontal 5HT2AR regulate the striatal activity via the modulation of cortical pyramidal neurons.

Such a scheme allows the synthesis of many of the disparate data that have been previously enumerated between the different neurotransmission systems. Thus, an integrated explanation for the occurrence of psychosis is proposed, with respect to the implication of the different aforementioned pharmacological systems. Nevertheless, this theory struggles to properly explain the subtle clinical dissimilarities that are observed depending on the underlying disorder or the ingested drug.

## 4. Discussion

Whether hallucinations occur in schizophrenia or after drug intoxication, they are very often clinically associated with a collection of other symptoms, including delusions, thought disorders, and loss of insight. All these symptoms are usually pooled into the general concept of psychosis. A particular issue about hallucinations is whether such a symptom can be nosologically distinguished from psychosis, or whether it is intrinsically linked in its occurrence with other psychotic symptoms such as delusions. Hallucinations are phenomenologically different from delusions, as hallucinations are misperceptions, while delusions are false beliefs. Nevertheless, both frequently appear mixed together or, if separated, are met in identical types of pathological states. Moreover, it has been contested that misperceptions and false beliefs rely on radically separated cognitive processes [60]. Dopaminergic theories suggest that both types of symptoms result from the increased dopaminergic transmission in the limbic striatum, even if there could be also subtle differences in their respective neural circuitry [2]. In this perspective, delusions and hallucinations are not separate clinical entities but nondissociable components of psychosis.

**Figure 2:**
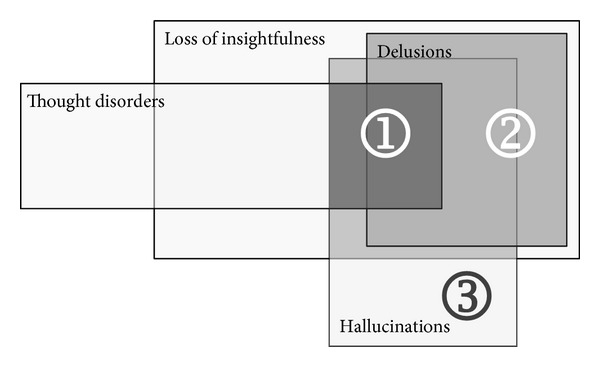
Different scopes of psychosis. The straightest definition of psychosis includes hallucinations or delusions with a loss of insight and thought disorders (1). A second definition is delusions or hallucinations with a sole loss of insightfulness (2). At last, several authors consider isolated hallucinations to belong to psychosis.

The first concern with that standpoint is that there are many definitions of what is psychosis (Figure 2) [61]. While thought disorders are sometimes considered to belong to psychotic symptoms, the most restrictive definition of psychosis is “delusions or prominent hallucinations in the absence of insight into their pathological nature” [61].

Moreover, whereas thought disorders are not always considered to be included in psychosis, situations in which hallucinations appear isolated from cognitive disorders are very rare. The state of consciousness is often clinically altered in some way. Even for hallucinations that occur in the general population, the state of consciousness appears to always be associated with infraclinical cognitive impairments in the executive functions and language abilities associated with the symptoms [62]. Thus, it appears difficult to affirm that hallucinations exist outside the scope of psychosis. Furthermore, a distinction has been proposed in the literature, between “hallucinations,” which would refer to “psychotic” states (i.e., associated with anxiety, disorganization and loss of control, and insight upon the symptoms [61]), and “pseudohallucinations” or “nonpsychotic hallucinations” [63], which refer to misperceptions with no anxiety and insightfulness that the misperceptions are not real [33]. This distinction deserves full attention because “pseudohallucinations” are frequent with psychedelics [33]. Additionally, they have not been reported with psychostimulants or dissociative anesthetics and are rare in acute schizophrenia either. It appears, however, that there could be a threshold effect with psychedelics, above which pseudohallucinations become vivid hallucinations, with loss of insight and increased anxiety [64]. Future studies should precise whether “pseudohallucinations” occur exclusively with 5HT2AR-related drugs or whether there is a dose-effect mechanism that underlies all types of hallucinogenic drugs. If the former scenario was true, it would imply that only psychedelics could induce misperceptions without psychosis, which would restrict such clinical patterns to the sole activity of the HT2ARs.

Increased insight does not appear to be the only feature of HT2AR-induced symptoms. The visual component of symptomatology appears to occupy a much larger place than in other hallucinatory pictures, particularly those observed in schizophrenic-related disorders. Furthermore, visual hallucinations occurring in Parkinson's disease have also been related to 5HT2ARs [65], and recent studies note that the serotoninergic system plays a central role in the visual processing [66]. The occurrence of synesthesia, which is almost uniquely observed with psychedelics and with other hallucinogenic drugs, is relevant to this hypothesis. It appears that, compared with other hallucinogenic drugs, only psychedelics impair the integrity of visual functioning. Consequently, it could be presumed that psychedelics do not trigger strictly the same types of neurobiological processes that are triggered by NMDAR antagonists or even dopaminergic drugs. However, there could be some overlapping, since a recent neuroimaging study has found that psychedelic activity may be related to a disruption in the network relating the prefrontal cortex with the posterior cingulate cortex [67]. Other investigations found that the same brain areas were similarly disrupted by NMDAR antagonist drugs [68, 69]. However, psilocybin-induced visual hallucinations and synesthesia have been repeatedly associated with occipitoparietal cortex activity [49, 66, 70], which has not been the case either for VH induced either by NDMAR antagonists, or for VH of schizophrenia [71] or first psychosis episode [72].

Nonperceptive symptoms induced by psychedelics are also very specific. These mystical feelings consist of a merging with the external world. This phenonenon, called “oceanic boundleness” [33], is not commonly reported with other classes of hallucinogenic drugs. We assume that the origin of such feelings is derived from a cognitive reconstruction following preliminary visual disruptions. Indeed, serotonergic synesthesia is defined as projections of nonvisual percepts onto the visual field. If one hypothesizes that both acoustic and kinesthetic information can be projected onto the visual field during psychedelic intoxication, then the subject could pathologically overlap sensations of corporal identity with visual perception and thus experience a feeling of merging with the outside world. Of course, such a presumption would require additional experimental support.

Even if all of the hallucinogenic drugs act by modulating the stimuli filtering and integrator system [28], it is also possible that each pharmacological system also specifically acts on other cerebral processes, which could confer quite a specific phenomenological pattern to the disruption of one system compared to the other. Thus, activation of 5HT2ARs could disrupt the information filtering system and induce at the same time a specific process of multisensory attraction by the visual system. On the other hand, NMDAR antagonists could disrupt the information filtering system, thereby enhancing the risk that hallucinations could appear, but at the same time cause interference in several cognitive processes and induce a loss of insight and social withdrawal. Lastly, dopaminergic stimulation may involve a specific dimension of excitement and motor agitation, as it is the main effect of psychostimulants drugs, which is not observed in other drug intoxications.

In conclusion, all three hallucinatory mechanisms—D2R activation, 5HT2AR activation, and NMDAR blockage—are proposed to trigger partial overlapping neurobiological processes whose hallucinations, among other psychotic symptoms, are the clinically expressed. In addition, the modulation of each of these three receptors induces characteristic cognitive impairments that give each class of hallucinatory drug a specific clinical tonality.

## Figures and Tables

**Table 1:** Characteristics of main pictures of hallucinations.

	Psychostimulants (cocaine, amphetamine)	Dissociative anesthetics (PCP, ketamine)	Psychedelics (LSD, psilocybin)	Schizophrenia (paranoid)
Supposed pharmacological mode of action	D2R activation	NMDAR blockage	5HT2AR activation	D2R activation/NMDAR blockage
Main type of hallucinations	Auditory	Visual	Visual	Auditory
Most frequent associated delusions	Paranoid	paranoid	Mystical	Paranoid
Most frequent associated behaviour	Agitation	Social withdrawal	Mystical feelings	Variable
Insightfulness	No	No	Yes	No
